# Toll-Like Receptor-4 Antagonist Enhances the Repair of Ultraviolet Radiation-Induced DNA Damage and Augments Anti-Tumor Immune Responses in Mice

**DOI:** 10.3390/cancers13215406

**Published:** 2021-10-28

**Authors:** Mohammad Asif Sherwani, Ahmed Abdelgawad, Minh Chung, Saad Ibrahim, Mualla Eraslan, Craig A. Elmets, Nabiha Yusuf

**Affiliations:** 1 Department of Dermatology, University of Alabama at Birmingham, 1670 University Boulevard, VH 566A, P.O. Box 202, Birmingham, AL 35294, USA; sherwani@uab.edu (M.A.S.); gawad@uab.edu (A.A.); mchung97@uab.edu (M.C.); sibrahim@uab.edu (S.I.); mualla@uab.edu (M.E.); celmets@uabmc.edu (C.A.E.); 2 Veteran Affairs Medical Center, Birmingham, AL 35294, USA; 3 O’Neal Comprehensive Cancer Center, University of Alabama at Birmingham, Birmingham, AL 35294, USA

**Keywords:** ultraviolet radiation, TLR4 inhibitor, skin cancer, DNA repair, immune response

## Abstract

**Simple Summary:**

Ultraviolet B (UVB) radiation is largely responsible for the development of skin cancer. When UVB-induced DNA damage in cells is not repaired, it can lead to the initiation of non-melanoma skin cancers. Xeroderma pigmentosum (XP) disease is caused by a defect in the repair of damaged DNA. Toll-like receptor-4 (TLR4) and NLR family pyrin domain containing 3 (NLRP3) belong to the family of innate immune receptors and are highly expressed in skin tumors. In this study, we determined the mechanism through which TLR4 inhibitor TAK-242 regulates inflammation and prevents skin cancer.

**Abstract:**

Ultraviolet (UV) irradiation of the skin is related to the development of skin cancer. UVB also causes DNA damage in the form of cyclobutane pyrimidine dimers (CPDs), which can result in stable mutations. Toll-like receptor 4 (TLR4), a component of innate immunity, plays a key role in cancer. Previous studies from our laboratory have observed that TLR4 deficiency resulted in the repair of UVB-induced DNA damage, inhibition of UVB-induced immune suppression, and carcinogenesis. In this study, we determined the efficacy of TLR4 antagonist TAK-242 in regulation of UVB-induced DNA damage, inflammation, and tumor development. Our results indicate that TAK-242 treatment increased the expression of xeroderma pigmentosum group A (XPA) mRNA, resulting in the repair of UVB-induced CPDs in skin of SKH-1 mice. Treatment with TAK-242 also inhibited the activation of NLR family pyrin domain containing 3 (NLRP3) in UVB-exposed skin of SKH-1 mice. Cutaneous carcinogenesis was significantly reduced in mice treated with TAK-242 in comparison to vehicle-treated mice. The proinflammatory cytokines IL-1β, IL-6, and TNF-α were also found to be significantly greater in vehicle-treated mice than TAK-242-treated mice. Finally, treatment with TAK-242 augmented anti-tumor immune responses in mice. Our data provide further evidence that activation of the TLR4 pathway promotes the development of UV-induced non-melanoma skin cancer mediated at least in part on its negative effects on DNA damage. Moreover, treatment with the TLR4 inhibitor TAK-242 may be effective for prevention of skin cancer.

## 1. Introduction

Ultraviolet (UV) B radiation is an important contributor to the growth and development of keratinocyte carcinoma (basal and squamous cell carcinoma) [1,2,3]. Although the predominant factor leading to UVB carcinogenesis is DNA damage, the role of inflammation and UVB-induced immunosuppression is also vital to its pathogenesis [4,5]. Every year, over 3.5 million new cases of non-melanoma skin cancer (NMSC) are diagnosed in the United States [6].

Toll-like receptors (TLRs) belong to a family of pattern recognition receptors (PRR), which play important role in innate immune responses [7]. Toll-like receptors are not only expressed on the cells of the immune system but are also expressed on tumor cells, where they may influence tumor growth and host immune [8]. Depending on the tumor type and/or the initiating agent, TLR4 ligation in the tumor microenvironment may have the beneficial effect of activating anti-tumor responses or the deleterious action of promoting non-specific inflammatory responses [9,10,11,12,13]. Polymorphisms in the TLR4 allele in humans have been linked to the cancer susceptibility and resistance, depending on the type of cancer studied. [14,15]. TLR4 is highly expressed in human skin tumors compared to normal skin [16,17]. However, there is limited information on the role TLR4 plays whether positive or negative effect on human keratinocyte carcinomas. In an animal model of UV carcinogenesis, we observed that TLR4 deficiency enhanced the repair of UVB-induced DNA damage in the form of cyclobutane pyrimidine dimers (CPD) in mouse and bone marrow dendritic cells (BMDC). Enhanced repair of DNA damage was associated with an increase in XPA mRNA expression [18]. We have also shown that loss of TLR4 resulted in prevention of UVB-induced suppression of immune responses by inhibiting development of regulatory T-cells [19]. We have shown that TLR4 deficiency inhibited UVB-induced chronic inflammation and reduced immune suppression in tumor microenvironment, resulting in enhanced tumor development [20]. In this study, we determined the role of TLR4 inhibitor, TAK-242, in the repair of DNA damage, inhibition of inflammation, and augmentation of cutaneous immune responses all of which contribute to prevention of photocarcinogenesis in mice.

## 2. Materials and Methods

Animals and reagents. SKH-1 mice were obtained from Jacksons Laboratories (Bar Harbor, ME, USA). XPA knockout mice were obtained from Dr Harry van Steeg (RIVM National Institute for Public Health and Environment, Blaricum, The Netherlands). Both male and female mice used for experiments were 6 to 8 weeks of age. All animal procedures were performed according to National Institute of Health (NIH) guidelines under protocols approved by the Institutional Animal Care and Use Committee of the University of Alabama at Birmingham. TAK 242 was procured from MedChem Express (Monmouth Junction, NJ, USA). ELISA kits (for IL-1β, IL-6 and TNF-α) were purchased from Invitrogen (Thermo Fisher Scientific, Waltham, MA, USA).

### 2.1. Antibodies

Monoclonal antibodies used for flow cytometry studies: anti-mouse CD16/CD32 (2.4G2; Purified), CD4 (RM4-5; AF700, PerCP-Cy5.5 and GK1.5; AF488), CD25 (PC61.5; APC, AF700), Foxp3 (FJK-16s; PE), IL-10 (JES5-16E3; PerCp), CD8 (53-6.7; PECy7, AF488), CD11b (M-170; PE, APC, and AF488), Gr-1(RB6-8C5; APC and PE) TNF-α (MP6-XT22; PE-Cy7) and IFNγ (XMG1.2; PE, APC) were purchased from Thermofisher Scientific (Waltham, MA, USA). Anti-CPD monoclonal antibody was purchased from Kamiya Biomedical Company (Seattle, WA, USA).

### 2.2. UVB Light Source and Irradiation of Mice

The UV source system (Daavlin, UVA/UVB Research Irradiation Unit, Bryan, OH, USA) was outfitted with a bank of four UVB lamps and an electronic controller to regulate UVB dosage at a fixed distance of 24 cm between the lamps and the dorsal skin surface of mice. Using Kodacel cellulose film, wavelengths less than 290 nm were filtered out (Eastman Kodak Co., Rochester, NY, USA). Most of the resulting wavelengths were in the UVB (290–320 nm; 80%) and UVA (20%) ranges, with peak emission at 314 nm as monitored on a regular basis. The dorsal skin of panels of wild type SKH-1 mice were exposed to a single dose of UVB radiation (100 mJ/cm^2^). C57BL/6 and XPA−/− mice were exposed to a single dose of 100 mJ/cm^2^ and 20 mJ/cm^2^ respectively for DNA damage experiments.

### 2.3. RNA Extraction and Real-Time PCR

Total RNA was extracted from skin and tumor samples using the Trizol reagent (Life Technologies, Carlsbad, CA, USA) according to manufacturer’s instructions. The iScript cDNA synthesis kit ((Bio-Rad, Hercules, CA, USA) was used to make cDNA from 1 µg RNA according to manufacturer’s instructions. Using iQ^TM^ SYBR Green Master Mix (Bio-Rad, Hercules, CA, USA), cDNA was amplified by real-time PCR with a Bio-Rad MyiQ thermocycler and SYBR Green detection system (Bio-Rad, Hercules, CA). The standard PCR was performed at 95 °C for 10 min, followed by 40 cycles at 95 °C for 30 s, 60 °C for 30 s, and 72 °C for 30 s. Custom primers (Table 1) were used to detect the expression of XPA, NLRP3, ASC, and caspase 1 and mRNA, which was then normalized to the expression level of GAPDH mRNA in each sample. In the case of mRNA analysis, the cycle threshold (Ct) procedure was used to measure the relative degree of gene expression. The expression of the target gene was calculated using the formulae 2**^−^**^ΔΔCT^ and the CT values from duplicate measurements, with normalization to a housekeeping gene used as an internal control.

### 2.4. Preparation of Tissue Lysates and Western Blot Analysis

For Western blot analysis of XPA protein in skin samples, lysates were prepared using 0.05 mL of RIPA buffer containing 20 mM HEPES, pH 7.4, 2 mM EDTA, 250 mM NaCl, 0.1% NP-40, 2 mg/mL leupeptin, 2 mg/mL aprotinin, 1 mM PMSF, 0.5 mg/mL benzamidine, 1 mM dithiothreitol, and 1 mM sodium vanadate. About 50–80 µg protein was loaded in each well and resolved on 10–12% SDS-polyacrylamide gel and transferred onto PVDF membranes. Membranes were incubated in blocking buffer for 1h and then incubated with the primary antibodies in blocking buffer for overnight at 4 °C. The membrane was then washed with TBS-T and incubated with secondary antibody conjugated with horseradish peroxidase. Protein bands were visualized using the enhanced chemiluminescence detection system (iBright Western Blot imaging systems, Thermofisher Scientific, Waltham, MA, USA). To verify equal protein loading and transfer of proteins from gel to membrane, the blots were stripped and reprobed for β-actin [18,20]. The band density was analyzed using IMAGE J software provided by the NIH and the values were normalized to the β-actin band density. All the whole western blot figures can be found in the supplementary Figure S1

### 2.5. CPD Quantitation by ELISA

SKH-1 mice were irradiated with a single dose of 100 mJ/cm^2^ and their dorsal skin (1 cm^2^) was harvested at 30 m, 24 h, and 48 h post-UVB irradiation. Mice that were not exposed to UVB were used as controls. The skin tissue was washed, and genomic DNA was extracted using DNeasy Blood and Tissue kit (Qiagen, Germantown, MD, USA). CPDs were quantified using the STA-322 DNA damage ELISA kit (Cell Biolabs, San Diego, CA, USA) according to manufacturer’s protocol.

### 2.6. Detection of CPD+ Cells in Skin Sections

A protocol mentioned previously with some modifications [23] was used to detect UVB-induced DNA damage in the form of CPD+ cells. To denature nuclear DNA, paraffin embedded skin sections (5 μm thick) were thawed and held in 70 mM NaOH in 70% ethanol for 2 min, then neutralized for 1 min in 100 mM Tris-HCl (pH 7.5) in 70% ethanol. Before incubation with a monoclonal antibody specific for CPD (Kamiya Biomedical Company, Seattle, WA, USA) or its isotype control, the sections were washed with PBS buffer and incubated with 10% goat serum in PBS to avoid non-specific binding. Bound anti-CPD antibody was detected after incubation with goat anti-mouse IgG1 followed by peroxidase. Sections were then incubated with diaminobenzidine and peroxidase substrate for 5 min. CPD^+^ cells were counted with an Olympus BX41 microscope in randomly selected 5–6 different high-power fields.

### 2.7. Measurement of Cytokines

The dorsal skin of panels of SKH-1 mice (5 per group) was treated with 0.5% TAK-242 or acetone and exposed to a single dose of UVB radiation (100 mJ/cm^2^)^.^ TAK-242 or acetone was applied topically to the dorsal skin of SKH-1 mice 30 min before exposure to UVB radiation. Serum was collected from five mice from each group 24 h after UVB exposure and the concentrations of IL-1β, TNF-α and IL-6 were determined using enzyme-linked immunosorbent assay (ELISA) kits (Thermofisher Scientific, Waltham, MA, USA) according to the manufacturer’s instructions.

### 2.8. Photocarcinogenesis Study

The dorsal skin of panels of SKH-1 (10 mice per group) was treated with 0.5% TAK-242 or acetone and exposed to UVB radiation (180 mJ/cm^2^), thrice a week up to 30 weeks.TAK-242 or vehicle was applied topically to the dorsal skin of SKH-1 mice 30 min before each exposure to UVB radiation. Mice were monitored for tumors on a weekly basis. In all experiments, mice that were not exposed to UVB were used as controls [20,24].

### 2.9. Flow Cytometry Analysis

After UVB exposure, the draining lymph nodes and spleens of panels of mice were harvested, and single cell suspensions were prepared according to the published protocol [25]. After 30 weeks of final UVB exposure, spleens, lymph nodes, and tumors were collected from panels of mice, and single cell suspensions of cells were prepared. Cells were stained for CD4, CD8, Foxp3, CD11b, Gr1, IL-10, TGF-β, and IFNγ (Thermofisher Scientific, Waltham, MA, USA). The percentage of cells was analyzed using flow cytometry (Attune NxT, Thermofisher Scientific, Waltham, MA, USA). The analysis was performed using FlowJo software version 10.6.1.

### 2.10. Statistical Analysis

UVB-exposed and non-exposed groups were compared separately in all experiments using one-way analysis of variance (ANOVA). The means and standard deviations are shown for all quantitative results. In each case a value of *p* < 0.05 was considered to be statistically significant.

## 3. Results

### 3.1. TAK-242 Treatment Repairs UVB-Induced CPDs

**Figure 1:**
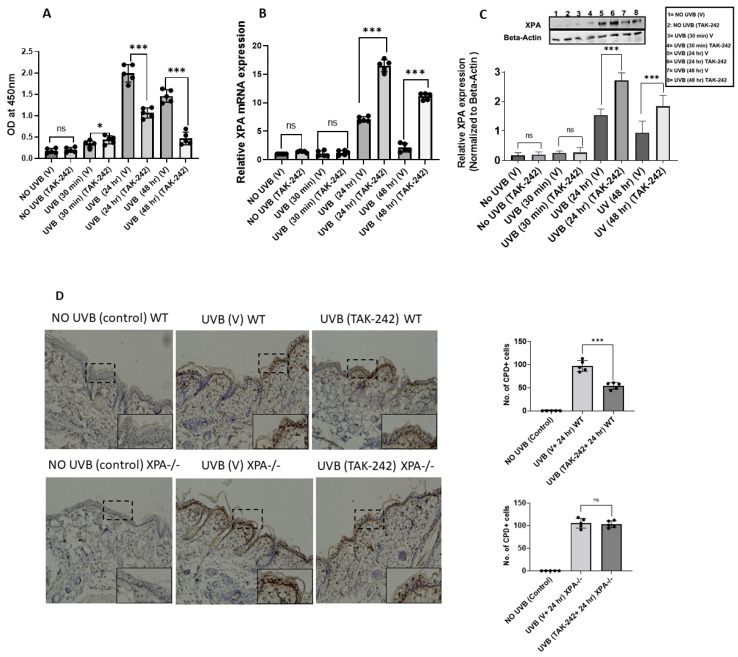

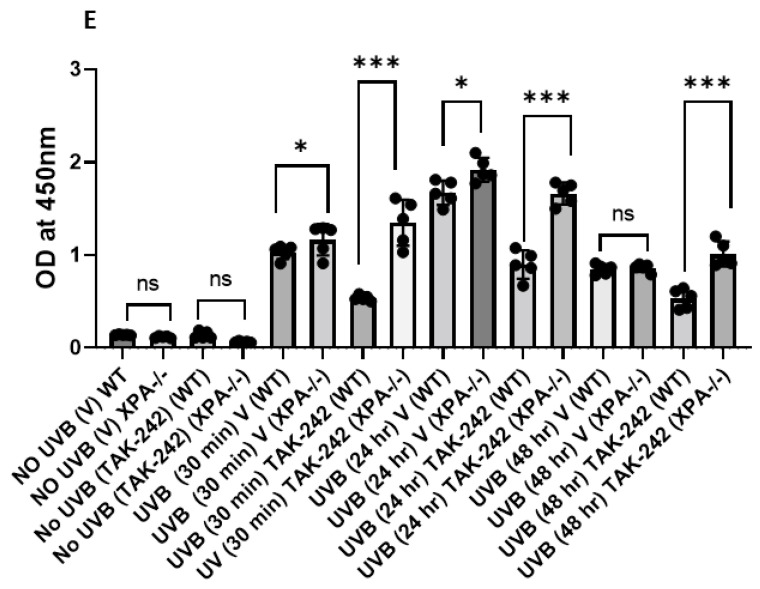
TLR4 inhibitor TAK-242 enhances the repair of UVB-induced CPDs. SKH-1 mice were exposed to a single dose of UVB radiation (100 mJ/cm^2^), and were thereafter sacrificed at different time points. Mice not exposed to UVB were used as controls. (**A**) DNA was also isolated from skin and subjected to ELISA for CPDs. There were fewer CPDs in the skin of TAK-242 treated WT mice. (**B**) Skin samples were also analyzed for expression of XPA mRNA. Expression of XPA mRNA was significantly higher in the skin of TAK-242 treated mice in comparison to vehicle-treated mice. (**C**) The Expression of XPA were further confirmed by Western blot assay. There was significantly higher expression of XPA protein in the skin of TAK-242 treated mice. (**D**) Panels of *XPA−/−* and WT (C57BL/6) mice were exposed to a single dose of UVB (100 mJ/cm^2^ for WT and 20 mJ/cm^2^ for *XPA*−/−) radiation. Formalin fixed skin sections (5 μM thick) were subjected to immunoperoxidase staining to detect CPD+ cells that stained brown. There were significantly fewer CPDs at 24 h in the skin of TAK-242 treated mice. CPD+ cells were not detected in non-UVB- exposed skin. There was no significant reduction in CPDs in the skin of XPA−/− mice. Magnification ×40. Summary of CPD+ cells. The number of CPD+ cells were counted in five to six different areas of the sections under an Olympus BX41 microscope. The numbers reported represent the CPD+ cells ± SD in epidermis. Scale bar = 50 μM. (**E**) DNA was also isolated from skin at 30 min, 24 h, and 48 h post-UVB exposure and subjected to ELISA for CPDs. There were fewer CPDs in the skin of TAK-242 treated WT mice. There was significant reduction in CPDs in *WT* mice treated with TAK-242. Experiments were conducted and repeated separately in 5 mice in each group with identical results. (* *p* < 0.05 and *** *p* < 0.001 and ns, not significant). The whole western blot figures of Figure 1C can be found in Figure S1.

UVB causes DNA damage in the form of CPDs, which are removed by the nucleotide excision repair (NER) [18]. To determine whether TAK-242 repairs UVB-induced DNA damage, panels of mice treated with vehicle or TAK-242 were exposed to UVB (100 mJ/cm^2^). Mice that were treated with vehicle or TAK-242 and not exposed to UVB, served as controls as mentioned in the Methods section. There were fewer UVB-induced CPDs in TAK-242 treated mice at various time points in comparison to vehicle-treated mice (Figure 1A). The expression of XPA mRNA was significantly higher in the UVB-exposed skin of TAK-242 treated mice at various time points, compared to the vehicle-treated group (Figure 1B). The protein expression of XPA was also significantly higher in the UVB-exposed skin of TAK-242 treated mice in comparison to vehicle-treated mice (Figure 1C).

To confirm whether TAK-242 treatment repaired CPDs, we examined CPDs in WT and XPA−/− mice by ELISA. As shown in Figure 1D, UVB-induced CPDs were significantly reduced after TAK-242 treatment at various time points. TAK-242 treatment did not affect CPDs in XPA−/− mice. The number of CPDs were further confirmed by immunohistochemistry method (Figure 1E).

### 3.2. TLR4 Inhibitor TAK-242 Inhibits UVB-Induced Activation of NLRP3 Inflammasome

**Figure 2:**
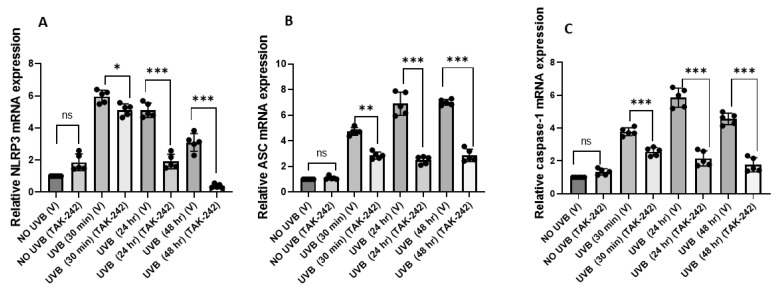
TLR4 inhibitor TAK-242 inhibits UVB-induced *NLRP3*, *Caspase-1*, and *ASC* activation. SKH-1 mice were exposed to a single dose of UVB radiation (100 mJ/cm^2^) and were thereafter sacrificed at different time points. Skin samples were obtained and analyzed for, NLRP3, caspase-1, and ASC mRNA expression. (**A**–**C**) The expression of NLRP3, Caspase-1, and ASC were significantly lower in the skin of TAK-242 treated mice. The expression level of *NLRP3*, *Caspase-1* and *ASC* mRNA was normalized to the expression level of the GAPDH mRNA in each sample as described in the Methods section. Experiments were conducted and repeated separately in 5 mice in each group with identical results. (* *p* < 0.05, ** *p* < 0.01, and *** *p* < 0.001 and ns, not significant).

UVB exposure causes activation of NLRP3 inflammasome via ASC and caspase-1 [26]. There is a crosstalk between TLR4 and NLRP3 and this amplifies inflammation [27]. To confirm whether TAK-242 inhibited the NLRP3 inflammasome, SKH-1 mice treated with TAK-242 or vehicle, were exposed to UVB. Mice were sacrificed at 30 min, 24 h, and 48 h to assess the expression of NLRP3, ASC, and caspase-1 by qPCR using custom primers. We found that TAK-242 treatment inhibited the expression of NLRP3, ASC, and caspase-1 mRNA in the skin of mice (Figure 2A–C).

### 3.3. TLR4 Inhibitor TAK-242 Inhibits UVB-Induced Inflammation

**Figure 3:**
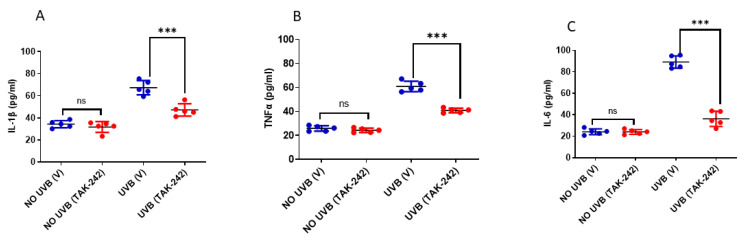
TLR4 inhibitor TAK-242 causes a decrease in expression of inflammatory cytokines. SKH-1 mice were exposed to a single dose of UVB radiation (100 mJ/cm^2^) and were thereafter sacrificed after 24 h to collect blood. Serum was collected from blood and concentrations of IL-1β, TNF-α and IL-6 were determined using Invitrogen (Thermofisher Scientific, Waltham, MA, USA) enzyme-linked immunosorbent assay (ELISA) kits according to the manufacturer’s instructions. (**A**–**C**) The levels of IL-1β, TNF-α and IL-6 were significantly reduced in serum, following treatment with TAK-242 followed by UVB exposure. The data represents mean ± SD from five mice. (*** *p* < 0.001 and ns, not significant).

IL-1β, IL-6 and TNF-α are known proinflammatory cytokines and are significantly elevated after UVB exposure [28]. We estimated the levels of these inflammatory cytokines in serum collected from the mice treated with TAK-242, 24h post-UVB exposure. We found that the serum levels of IL-1β, IL-6 and TNFα were significantly decreased in mice treated with TAK-242 in comparison to the vehicle-treated mice following UVB exposure (Figure 3).

### 3.4. TAK-242 Inhibits UVB-Induced Tumor Development

**Figure 4:**
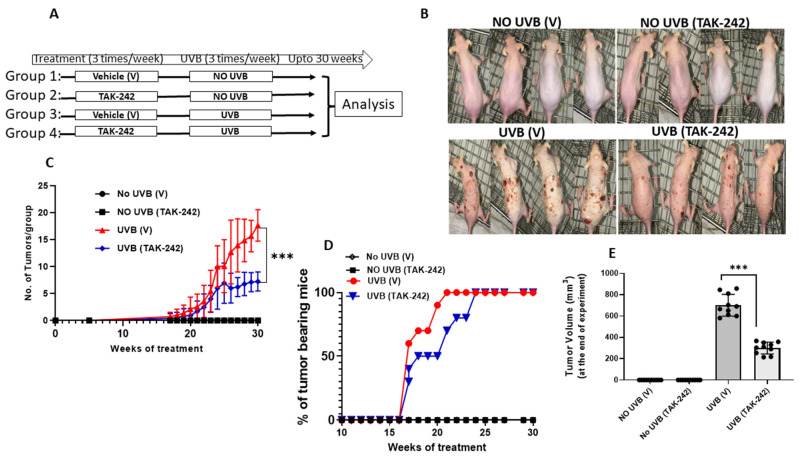
Topical treatment with TLR4 inhibitor TAK-242 blocks UVB-Induced tumorigenesis in mice. (**A**) Treatment scheme: The dorsal skin of panels of SKH-1 was treated with 0.5% TAK-242 (in acetone) or vehicle exposed to UVB radiation (180 mJ/cm^2^), thrice a week up to 30 weeks.TAK-242 or vehicle was applied topically to the dorsal skin of SKH-1 mice 30 min before each exposure to UVB radiation. Tumor numbers were recorded weekly until the end of experiment at week 30. (**B**) Appearance of mice at termination of experiment; (**C**) number of tumors per group; (**D**)percentages of tumor bearing mice; tumor volume at week 30 (**E**) At sacrifice, the TAK-242 treated group displayed a significant reduction in average tumor burden versus the vehicle control group. Experiments were conducted in 10 mice in each group. (*** *p* < 0.001).

To evaluate if topical application of TAK-242 inhibited UVB-induced tumor development, we subjected SKH-1 mice to a cutaneous photocarcinogenesis protocol [24]. SKH-1 mice were exposed to chronic doses of UVB radiation (180 mJ/cm^2^, thrice a week) for 30 weeks (Figure 4A). Tumor latency was 17 weeks in both groups, after initial exposure to UVB. There was a significant decrease in the number of tumors per group in the TAK-242 treated mice compared to the vehicle-treated animals beginning at 17 weeks, which plateaued through 30 weeks when the experiment was terminated (Figure 4B). At 21 weeks, 100% mice in the vehicle-treated group had tumors in comparison to TAK-242 group where 70% mice had tumors. By 25 weeks, mice in both TAK-242 and vehicle-treated group had tumors (Figure 4C). Mice in the TAK-242 group also had significantly less tumor volume in comparison to the vehicle-treated group (Figure 4D).

### 3.5. TAK-242 Inhibits the Generation of CD4+CD25+ Regulatory T-Cells

**Figure 5:**
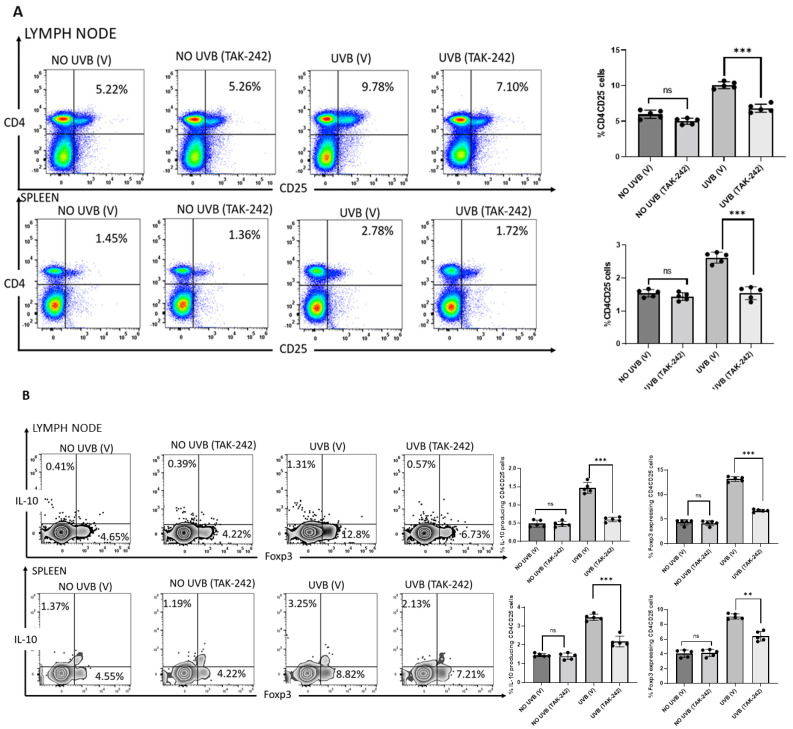
Generation of CD4+CD25+ regulatory T-cells Inhibited by TAK-242. SKH-1 mice were subjected to photocarcinogenesis experiment as mentioned in the Methods section. Mice were sacrificed at 30 weeks, and single cell suspensions of cells were prepared from the spleen and lymph nodes. Anti-CD4 and anti-CD25 antibodies were used to stain the cells, followed by anti-IL-10 and Foxp3 antibodies. Flow cytometry was used to count the number of CD4+CD25+ cells. (**A**) There were significantly fewer CD4+CD25+ regulatory T-cells in the spleens and lymph nodes of TLR4 inhibitor TAK-242 treated mice than in the vehicle-treated mice. The histograms depict the mean ± SD of cell percentages per group. Mice not exposed to UVB radiation were used as controls. (**B**) The expression of Foxp3 and IL-10 was also determined in CD4+CD25+ gated cells. There was significantly less Foxp3 and IL-10 in the TAK-242 treated mice. The histogram depicts the mean ± SD of Foxp3 and IL-10 expressing CD4+CD25+ cells per group. Experiments were conducted in 5 mice in each group. (** *p* < 0.01, *** *p* < 0.001 and ns, not significant).

We previously have found that animals deficient in TLR4 develop significantly fewer CD4+CD25+ regulatory cells following chronic UVB exposure than mice with normal TLR4 expression [20]. We examined whether TAK-242 treatment had a similar effect. We found that chronic doses of UVB (180 mJ/cm^2^) exposure significantly increased CD4+CD25+ regulatory T-cells in spleen and lymph nodes of vehicle-treated mice compared to non-exposed mice. After 30 weeks of exposure, this increase in CD4+CD25+ regulatory T-cells was significantly reduced in UVB-exposed tumor bearing mice treated with TAK-242 compared to UVB-exposed mice treated with vehicle after 30 weeks of exposure (Figure 5A). Furthermore, expression of Foxp3 and IL-10 was reduced in CD4+CD25+ gated cells isolated from tumor bearing mice treated with TAK-242 in comparison to vehicle-treated controls (Figure 5B).

### 3.6. TAK-242 Inhibits UVB-Induced Generation of CD11b+Gr1+ Myeloid Cells

**Figure 6:**
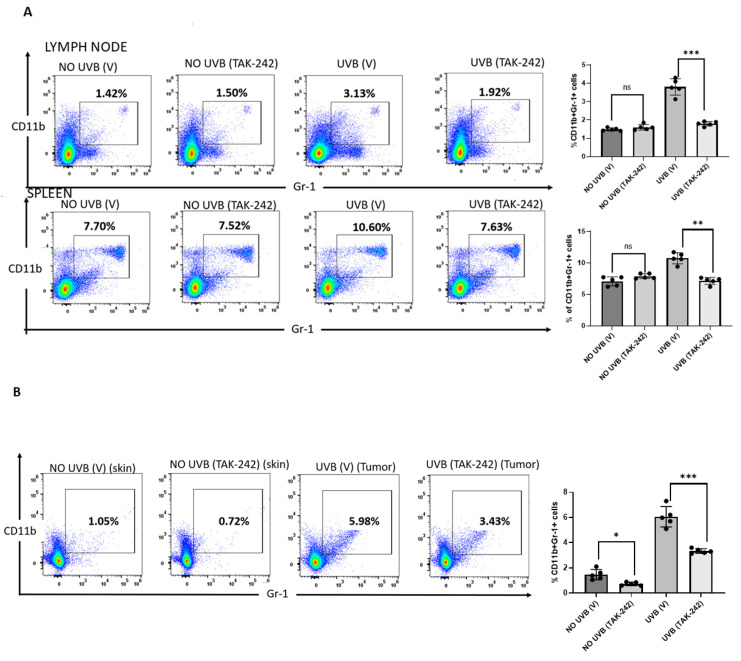
TAK-242 prevented the generation of CD11b+Gr1+ myeloid cells. SKH-1 mice were subjected to photocarcinogenesis experiment as mentioned in the Methods section. Mice were sacrificed at 30 weeks, and single cell suspensions of cells were prepared from the spleen and lymph nodes. Anti-CD11b and anti-Gr1 antibodies were used to stain the cells. The number of CD11b+Gr1+ cells, was analyzed by flow cytometry. There were significantly fewer Gr1+CD11b+ myeloid cells (** *p* < 0.01) in the spleens, (**A**) lymph nodes and (**B**) tumor of TAK-242 treated mice than vehicle-treated mice. The histograms depict the mean ± SD of cell number per group. Mice not exposed to UVB radiation were used as controls. Experiments were conducted in 5 mice in each group. (* *p* < 0.05, ** *p* < 0.01, *** *p* < 0.001 and ns, not significant).

Studies from our laboratory have shown that absence of TLR4 inhibited the generation of CD11b+Gr1+ myeloid cells in mice [20]. We found that chronic doses of UVB (180 mJ/cm^2^) exposure significantly increased CD11b+Gr1+ myeloid cells in the spleen and lymph nodes of vehicle-treated mice compared to unexposed mice. Topical application of TAK-242 significantly decreased the CD11b+Gr1+ myeloid cells in spleen and lymph nodes compared to CD11b+Gr1+ myeloid cells from vehicle-treated mice (Figure 6A). The percentages of Gr1+CD11b+ myeloid cells from tumors collected from mice treated with TAK-242 were significantly lower in comparison to the tumors collected from vehicle-treated mice (Figure 6B).

## 4. Discussion

Ultraviolet (UV) B is an environmental carcinogen and an immunosuppressant. It can modulate both innate and adaptive immune responses through multiple pathways and mechanisms [29]. Apart from being present on immune cells, it is expressed by many tumors including skin malignancies [9,10,11,12,13,16,17]. TLR4 expression has been found to be higher in cutaneous SCCs than in normal skin [17]. Expression of TLR4 progressively increases in in sun-damaged skin and actinic keratosis from patient matched samples [17]. TLR4 mediated carcinogenesis was dependent on both immune cells and radio resistant non-immune cells, possibly due to non-specific inflammation caused by over activation of TLR4 [30]. In contrast to UV carcinogenesis in which TLR4 deficient mice are resistant to skin tumor development, TLR4 deficient mice are more prone to chemically induced carcinogenesis. In that model, mice that develop contact hypersensitivity to the chemical carcinogen that initiates skin tumorigenesis [11]. We have recently reported that UVB-induced cutaneous carcinogenesis is retarded in TLR4 deficient mice relative to TLR4 proficient mice, and this occurs at least in part via inhibition of suppressor cells that augment pro-tumor responses [20]. It was also associated with a reduction in the development of regulatory T-cells that produce IL-10 and increased numbers of MDSCs in the tumor microenvironment [20]. DNA damage-repair pathways prevent UV carcinogenesis. Evidence that this is the case is derived from observations made in patients with xeroderma pigmentosum, an inherited disease in which there are mutations in the DNA damage-repair pathway. Individuals with this disease develop large numbers of skin cancers at an unusually early age. Immunological defects in adaptive immune responses have been identified in this disease. A recent study reported the role of TLR4 antagonist TAK-242 in prevention of photocarcinogenesis by down-regulation of inflammatory mediators and MAP kinase phosphorylation [24]. In this study, we determined the efficacy of TLR4 antagonist TAK-242 in regulation of UVB-induced DNA damage, inflammation, and tumor development. Our results indicate that TAK-242 treatment increases the expression of XPA resulting in the repair of UVB-induced CPDs in skin of SKH-1 mice.

The NLRP3 inflammasome is comprised of a sensor (NLRP3), an adaptor (ASC) and an effector (caspase 1). Activation of the inflammasome is a two-step process that involves priming, which occurs through recognition of pattern recognition receptors (PRRs) such as TLRs. The priming step is followed by an activator, which provokes full activation of the inflammasome [31]. We, and others have reported that UVB exposure can cause activation of the NLRP3 inflammasome via ASC and caspase-1 [26]. In this study, we found that treatment with TAK-242 inhibited the activation of the NLRP3 inflammasome in UVB-exposed skin of SKH-1 mice.

When SKH-1 mice were exposed to multiple doses of UVB radiation (180 mJ/cm^2^) for 30 weeks, cutaneous carcinogenesis was significantly retarded in mice treated with TAK-242 in comparison to vehicle-treated mice. These results are consistent with the study by Blohm-Mangone et al., in which they showed that TAK-242 blocks UV-induced activation of NF-kB and AP-1 signaling that is accompanied by down-regulation of inflammatory mediators and phosphorylation of MAP kinase [32]. We also found that the proinflammatory cytokines IL-1β, IL-6, and TNF-α were significantly down-regulated in TAK-242 treated mice.

Tumors express neoantigens that should be able to generate an immune response that recognizes and eradicates cancer cells. There are various mechanisms that suppress anti-tumor immunity [33,34,35]. Chronic inflammation is one of the key factors that cause immune suppression [36]. One of the mechanisms through which inflammation leads to immune suppression is by recruitment of CD11b+Gr1+ myeloid derived suppressor cells (MDSC) into the tumor microenvironment [33,34]. CD11b+Gr1+ cells are well known to have a suppressive effect on anti-tumor immune responses [34,35,36,37,38]. Mice exposed to UVB produce have been reported to produce CD11b+Gr1+ myeloid cells [39,40]. TLR4 deficiency results in inhibition of CD11b+Gr1+ cells generated after chronic exposure to UVB [20]. Consistent with our previous studies, we found that chronic doses of UVB exposure significantly increased the generation of CD11b+Gr1+ myeloid cells in spleen, lymph nodes, and tumors in mice. This increase in CD11b+Gr1+ myeloid cells was significantly reduced in UVB-exposed mice that were treated with the TLR4 inhibitor.

UVB-induced regulatory T-cells play an important role during photocarcinogenesis, due to their ability to inhibit anti-tumoral effector functions [41,42]. Studies have shown that CD4+ UVB-induced regulatory T-cells are involved in the inhibition of effector cells during the development of UV-induced immunotolerance of skin tumors [43,44]. CD4+CD25+Foxp3+ T-cells can be found intermingled with human basal cell carcinoma along with the TH_2_ cytokines IL-4 and IL-10 [45].

We found that TLR4 deficiency resulted in inhibition of CD4+CD25+ cells generated after chronic UVB exposure [20]. We also observed that chronic doses of UVB exposure significantly increased the splenic CD4+CD25+Foxp3+ regulatory T-cells in comparison to unexposed mice. This increase in CD4+CD25+Foxp3+ regulatory T-cells was significantly reduced in UVB-exposed, TLR4 inhibitor-treated mice. These findings provide additional support for the deleterious effects of TLR4 on UVB radiation-induced cutaneous carcinogenesis, and are consistent with a scenario in which both keratinocytes and cutaneous immunity are affected by its inhibitory effect on DNA repair. In keratinocytes, TLR4 inhibits the repair of CPDs, the initiating event in UVB-induced cutaneous carcinogenesis. With respect to cutaneous immunity, the failure to repair damaged DNA leads to the generation of CD4+CD25+ regulatory T-cells and recruitment of MDSCs into the tumor microenvironment, both of which serve to limit host defenses directed at cells destined to become skin tumors. At the same time, TLR4 ligation enhances non-specific inflammatory responses through the NLRP3 inflammasome and production of proinflammatory cytokines that promote UVB-induced carcinogenesis.

The epidemic of skin cancer represents a major public health issue and is a tremendous cost to healthcare systems in the United States and worldwide [46,47]. There is a need for effective preventive strategies for management of skin cancer. Our studies, in the past decade, on the role of TLR4 in regulation of UVB-induced DNA damage, immune suppression and tumor development have paved the way for pre-clinical studies in this area from our group and others [20,24]. In the present study and those of others, TLR4 antagonist, TAK-242 was effective in prevention of UVB-induced skin cancer in animal models and pave the way for clinical trials in which TAK-242 is evaluated for prevention of UV-induced skin cancer.

## 5. Conclusions

Resatorvid (TAK-242) is a TLR4-sepcific pharmacological inhibitor that is being tested in clinical trials. We and others have shown that TAK-242 has been effective in inhibition of TLR4 in skin cells and mice after single exposure to UVB radiation. We have found that expression of NLRP3 is also reduced in skin cells treated with TAK-242. There is no information on the role of TAK-242 in DNA damage and prevention of UVB-induced skin tumors. In the present study, we addressed the mechanism elicited by which TLR4 inhibitor TAK-242 prevent the development of UVB-induced skin tumors. We have shown how TAK-242 highly effective in preventing UVB-induced non-melanoma skin cancer in mice by the repair of UVB-induced DNA damage and inactivation of NLRP3 inflammasome in the skin.

## Figures and Tables

**Table 1:** Primer sequences used in reverse transcription–polymerase chain reaction.

Gene	Primer Sequence	References
GAPDH	5′-AACTTTGGCATTGTGGAAGG-3′ 5′-ACACATTGGGGGTAGGAACA-3′	[18]
XPA	5′-CAAAGGTGGCTTCATTTTAG-3′ 5′-GGTACATGTCATCTTCTAAG-3′	[18]
NLRP3	5′-ATTACCCGCCCGAGAAAGG-3′ 5′-TCGCAGCAAAGATCCACACAG-3′	[21]
Caspase-1	5′-GGAAGCAATTTATCAACTCAGTG-3′ 5′-GCCTTGTCCATAGCAGTAATG-3′	[22]
ASC	5′-CAGCAACACTCCGGTCAG-3′ 5′-AGCTGGCTTTTCGTATATTGTG-3′	[22]

## Data Availability

The data presented in this study are available on request from the corresponding author.

## Supplementary Materials

The following are available online at https://www.mdpi.com/article/10.3390/cancers13215406/s1, Figure S1: the whole western blot figures of Figure 1C.
