# Supplementary figures and images for: Toll-Like Receptor-4 Antagonist Enhances the Repair of Ultraviolet Radiation-Induced DNA Damage and Augments Anti-Tumor Immune Responses in Mice
Source: Cancers (Basel). 2021 Oct 28;13(21):5406. doi: 10.3390/cancers13215406 (PMC8582386; doi:10.3390/cancers13215406)

## Slide 1
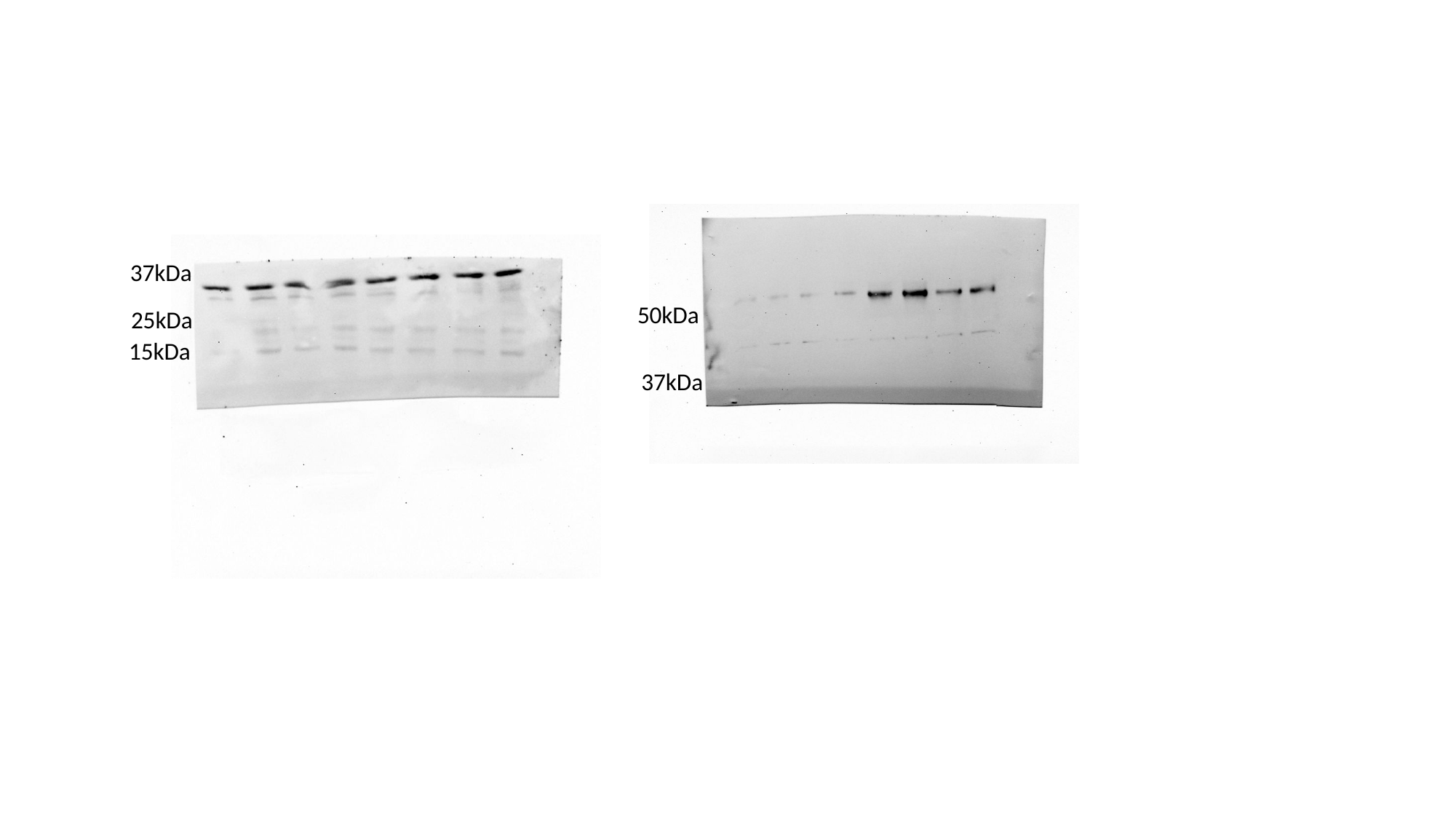

37kDa
50kDa
25kDa
15kDa
37kDa

Supplement: Supplementary file 1 [file cancers-13-05406-s001.zip › cancers-1371990-supplementary materials.pptx]
